# Use of mesoporous polydopamine nanoparticles as a stable drug-release system alleviates inflammation in knee osteoarthritis

**DOI:** 10.1063/5.0088447

**Published:** 2022-04-21

**Authors:** Yun Wang, Weiwen Ge, Zhigui Ma, Guangyu Ji, Mingsong Wang, Guangdong Zhou, Xiansong Wang

**Affiliations:** 1 Department of Plastic and Reconstructive Surgery, Shanghai Ninth People's Hospital, Shanghai Jiao Tong University School of Medicine, Shanghai Key Laboratory of Tissue Engineering, Shanghai 200011, China; 2 Department of Oral and Craniomaxillofacial Surgery, Shanghai Ninth People’s Hospital, Shanghai Jiao Tong University School of Medicine; Shanghai Key Laboratory of Stomatology & Shanghai Research Institute of Stomatology, National Clinical Research Center for Oral Diseases, Shanghai 200011, China; 3 Department of Oral Surgery, Shanghai Ninth People's Hospital, Shanghai Jiao Tong University School of Medicine, Shanghai Key Laboratory of Stomatology, Shanghai 200011, China; 4 Department of Thoracic Surgery, Shanghai Ninth People's Hospital, Shanghai Jiao Tong University School of Medicine, Shanghai 200011, China

## Abstract

Osteoarthritis drugs are often short-acting; therefore, to enhance their efficacy, long-term, stable-release, drug-delivery systems are urgently needed. Mesoporous polydopamine (MPDA), a natural nanoparticle with excellent biocompatibility and a high loading capacity, synthesized via a self-aggregation-based method, is frequently used in tumor photothermal therapy. Here, we evaluated its efficiency as a sustained and controlled-release drug carrier and investigated its effectiveness in retarding drug clearance. To this end, we used MPDA as a controlled-release vector to design a drug-loaded microsphere system (RCGD423@MPDA) for osteoarthritis treatment, and thereafter, tested the efficacy of the system in a rat model of osteoarthritis. The results indicated that at an intermediate drug-loading dose, MPDA showed high drug retention. Furthermore, the microsphere system maintained controlled drug release for over 28 days. Our *in vitro* experiments also showed that drug delivery using this microsphere system inhibited apoptosis-related cartilage degeneration, whereas MPDA-only administration did not show obvious cartilage degradation improvement effect. Results from an *in vivo* osteoarthritis model also confirmed that drug delivery via this microsphere system inhibited cartilage damage and proteoglycan loss more effectively than the non-vectored drug treatment. These findings suggest that MPDA may be effective as a controlled-release carrier for inhibiting the overall progression of osteoarthritis. Moreover, they provide insights into the selection of drug-clearance retarding vectors, highlighting the applicability of MPDA in this regard.

## INTRODUCTION

Osteoarthritis, which is the most common chronic degenerative disease of the joints in elderly individuals, is characterized by sliding membrane inflammation, cartilage loss, and osteophyte formation.^1^ Oral medication for osteoarthritis has adverse effects; however, joint cavity drug injection, which does not cause these adverse effects, is a highly effective clinical treatment strategy.^2,3^ Unfortunately, the drugs thus injected into the joint cavity are quickly removed by the lymphatic system, thus their efficacy is considerably reduced.^4,5^ In particular, the commonly used drugs for osteoarthritis treatment are short-acting and are therefore often administered repeatedly to relieve pain during long-term treatment. For instance, hyaluronic acid, which has been used to treat early osteoarthritis symptoms, is short-acting, indicating that multiple articular injections are often required within a given month. It also shows poor patient adaptability, and frequent injection increases the risk of knee infections.

Various drug carriers have been developed to ensure controlled and long-term drug delivery to treat osteoarthritis.^6,7^ However, most nanocarriers have limited biological applications. For example, liposomes, micelles, and dendrimers are typically low-load agents, whereas inorganic porous materials are toxic and insufficiently degradable.^8^ Owing to its safety, reliability, and low toxicity as well as its physical and chemical properties, polylactic acid is frequently used as a drug carrier in clinical studies.^9–12^ However, its use in this regard is associated with several limitations, including a low effective loading rate, rapid drug release, and acidic degradation product formation. These factors imply that polylactic acid use to achieve long-term controlled drug release is challenging.^13,14^ Thus, there is an urgent need for a long-term, stable-release drug-delivery system for the treatment of chronic degenerative diseases. For osteoarthritis, in particular, this system should enable drug release in the joint cavity; this is necessary to achieve more efficient and long-term treatment effects.

Mesoporous polydopamine (MPDA) is a nanoparticle that has demonstrated efficacy in the treatment of tumors via the photothermal effect.^15,16^ However, studies on its capacity to retard drug clearance are limited. Nevertheless, it offers specific advantages, including excellent biocompatibility, a high loading capacity, and a self-aggregation-based synthesis method.^15,17,18^ Furthermore, MPDA nanoparticles can be synthesized faster with more ease and using more economical strategies than other nanoparticles. Thus, they have potential for application as a controlled-release drug carrier.

**FIG. 1.**
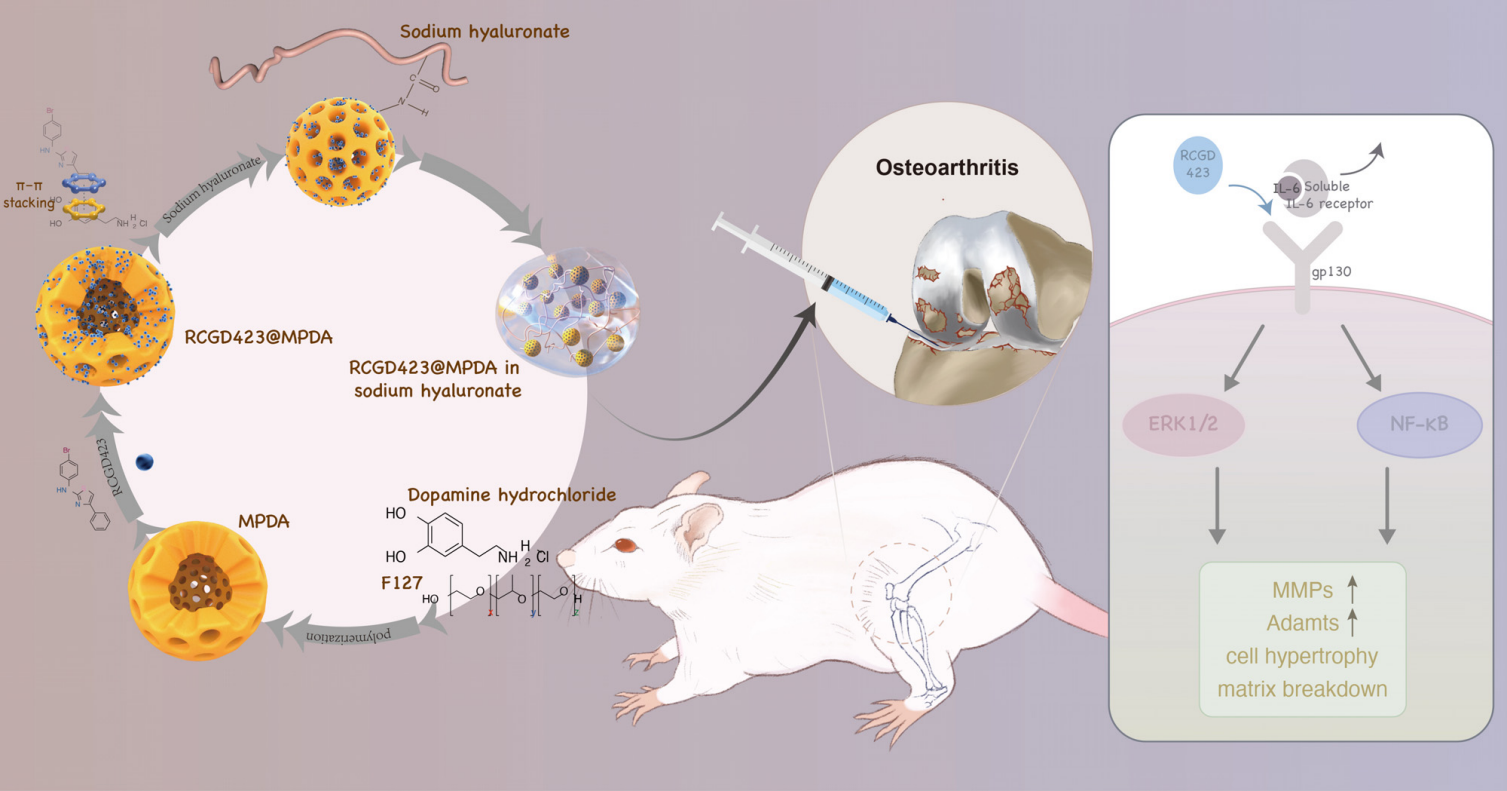
Schematic showing the preparation of RCGD423-loaded mesoporous polydopamine (MPDA) nanoparticles for alleviating osteoarthritic inflammation.

Based on its excellent biocompatibility and strong drug-carrying capacity, it can be considered that the microstructure of MPDA possibly confers a certain level of drug-retention and drug-release capabilities that allow sustained drug release. Therefore, we hypothesized that MPDA can facilitate sustained and controlled drug release, enabling the efficient and long-lasting treatment of osteoarthritis (Fig. 1). To verify this hypothesis, we prepared a microsphere system (RCGD423@MPDA) for osteoarthritis treatment with MPDA as the controlled-release vector. Thereafter, we evaluated drug loading and controlled release of this system as well as its capacity to regulate osteoarthritis-induced inflammation *in vitro* and *in vivo* (using a rat osteoarthritis model). Notably, RCGD423 is a small molecule that regulates interleukin-6 (IL-6)-induced inflammatory responses.^9^ We believe that the application of our findings could improve current treatment strategies for osteoarthritis and other chronic inflammatory diseases.

## RESULTS

### Characterization of MPDA nanoparticles

**FIG. 2.**
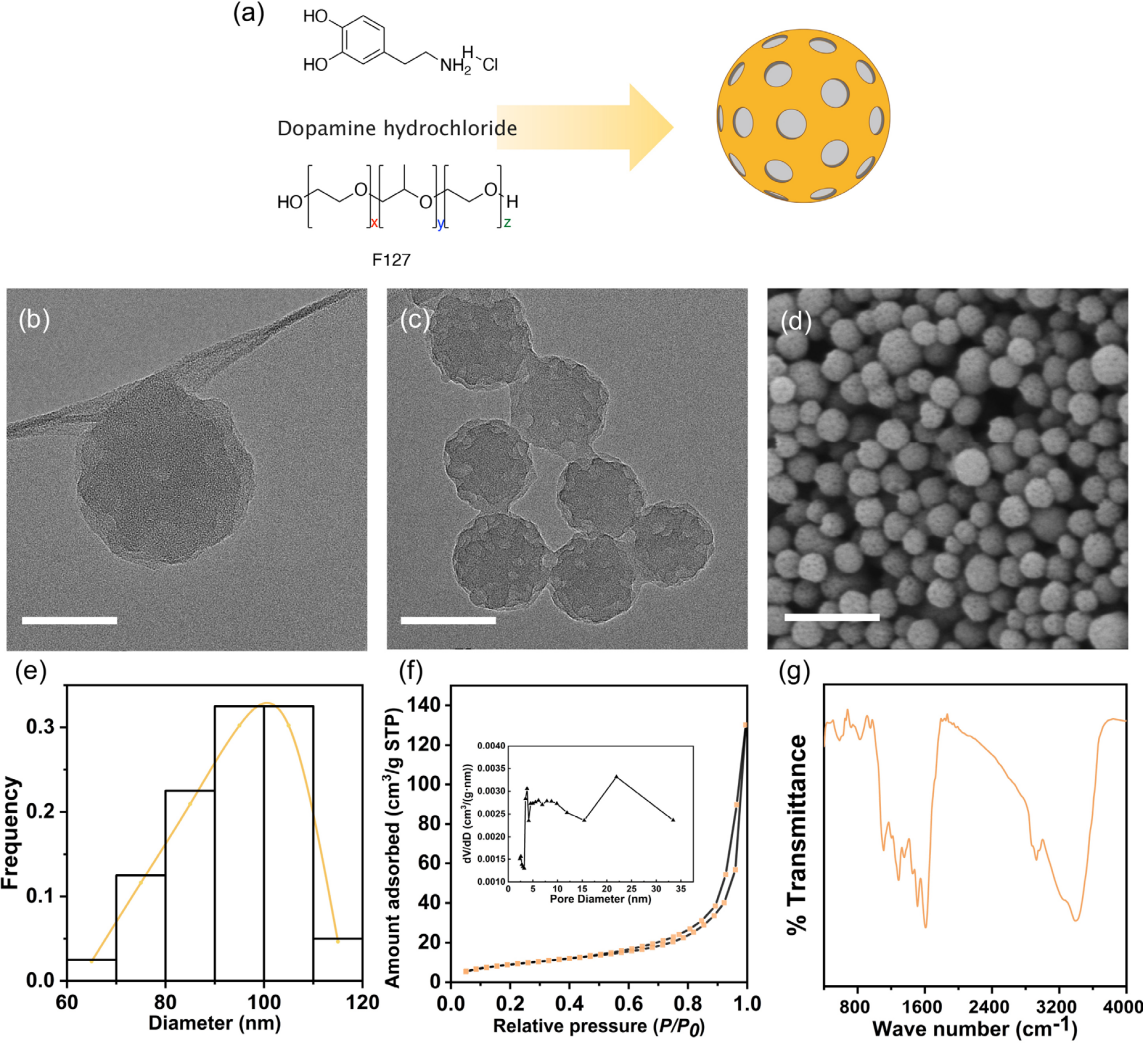
Characterization of mesoporous polydopamine (MPDA) nanoparticles. (a) Structural diagram. (b) TEM images; scale bar = 50 nm. (c) TEM images; scale bar = 100 nm. (d) SEM image; scale bar = 500 nm. (e) Pore size distribution on MPDA in terms of diameter (n = 43). (f) Nitrogen sorption isotherm and the corresponding pore size distribution deduced from the desorption branch of the isotherm. (g) Fourier transform-infrared spectrum of MPDA nanoparticles.

The synthesized MPDA nanoparticles are shown in Fig. 2(a), and the results of their analysis using transmission electron microscopy (TEM) [Figs. 2(b) and 2(c)] and SEM [Figs. 2(d) and 2(e)] revealed that the MPDA sample consisted of spherical particles with a mean diameter (SD) of 94.1 (11.6) nm. Furthermore, the MPDA molecules exhibited a prominent type-IV hysteresis loop structure [Fig. 2(f)], indicating a mesoporous structure; based on this type-IV hysteresis loop, we inferred that the observed mesoporosity was due to the stacking of layered plate-like structures. In addition, based on Brunauer–Emmett–Teller (BET) surface area ratio analysis, the specific surface area of MPDA was determined to be 34.5 m^2^/g, and based on its adsorption capacity under the maximum adsorption pressure, its pore volume was 0.20 cm^3^/g.

The analysis of pore size distribution of MPDA using desorption curve data revealed the predominance of mesopores, and the pore size distribution was found to be bimodal, with the mesopores of sizes predominantly distributed in the ranges of 2–5 and 15–35 nm. In the Fourier transform-infrared spectrum of MPDA [Fig. 2(g)], the peak 3391 cm^−1^ corresponded to O–H and N–H stretching vibrations, whereas those at 2929 and 1609 cm^−1^ corresponded to C–H stretching vibrations and aromatic ring-associated N–H bending vibrations, respectively. In addition, the peak at 1512 cm^−1^ corresponded to N–H bending vibrations and that at 1455 cm^−1^ corresponded to C–H turning vibrations. The peaks at 1355 and 1287 cm^−1^ corresponded to C–OH bending and stretching vibrations on the benzene ring, respectively, while the peak at 1110 cm^−1^ corresponded to C–O stretching vibrations.

### Fabrication and characterization of RCGD423@MPDA microspheres

**FIG. 3.**
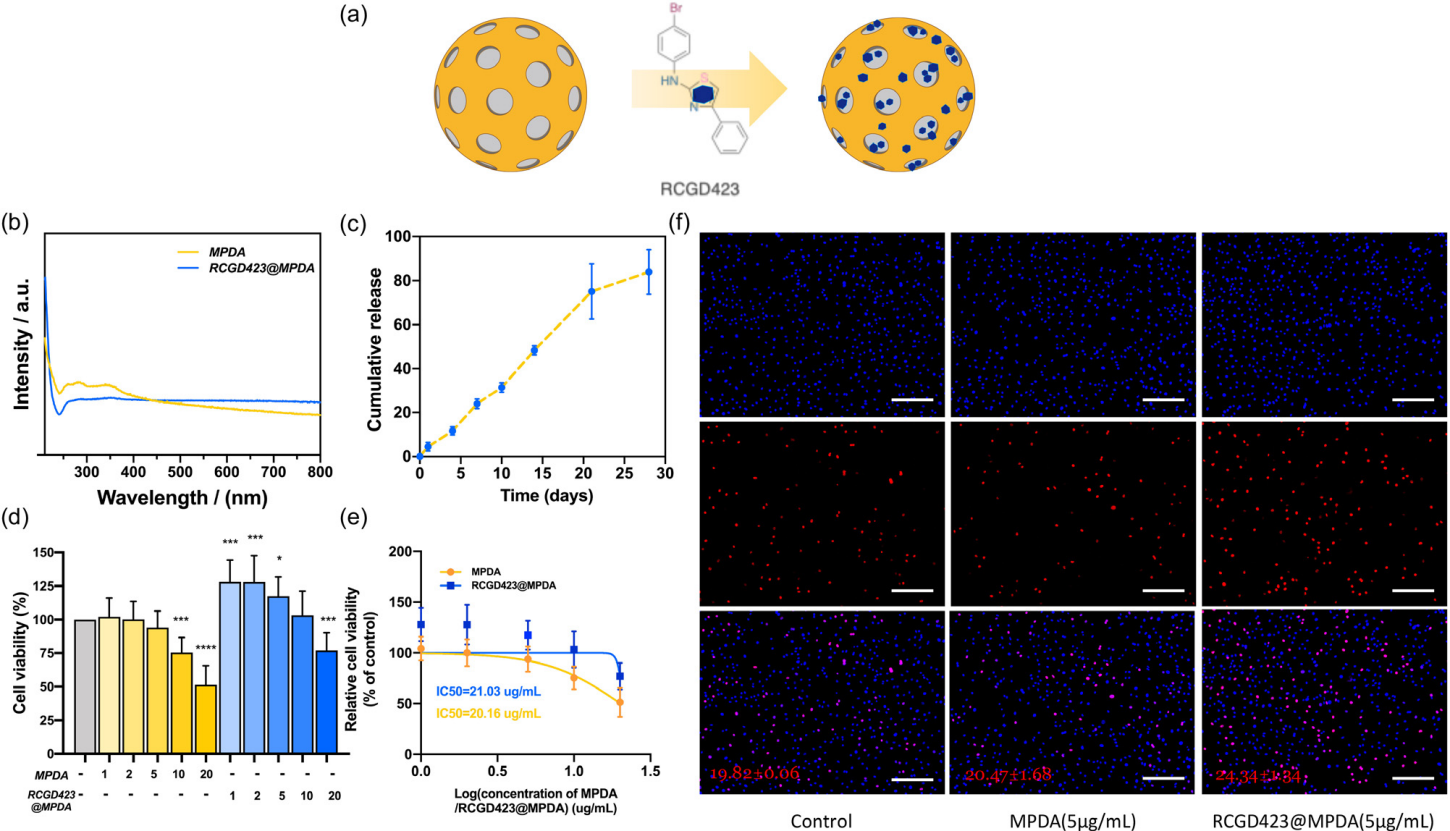
Characterization of RCGD423@MPDA particles. (a) Structure of RCGD423 loading on mesoporous polydopamine (MPDA) nanoparticles. (b) Comparison of the UV-visible absorption spectra of MPDA and RCGD423@MPDA in PBS. (c) Release profile of RCGD423 from 0.5 mg of RCGD423@MPDA nanoparticles in PBS over a 28-day period. (d) Images of chondrocytes treated with RCGD423@MPDA nanoparticles, compared with untreated (control) and MPDA-only treated chondrocytes on day 2. (e) Viability of MPDA-only or RCGD423@MPDA-treated chondrocytes, relative to the control; analysis was performed using GraphPad Prism software. (f) Chondrocytes treated with RCGD423@MPDA nanoparticles for 24 h. The chondrocytes were EdU-stained; red staining represents proliferating cells. Scale bar = 250 *μ*m. *P < 0.05, **P < 0.01, ***P < 0.001, and ****P < 0.0001, vs control.

Next, we loaded RCGD423 (400 *μ*g/ml in PBS; pH = 7.4) onto MPDA [Fig. 3(a)] and assessed its adsorption by MPDA. We observed that the drug content in the supernatant after 48 h was 14%, indicating that 86% of the RCGD423 feed was adsorbed on MPDA. Thus, RCGD423@MPDA, at an intermediate RCGD423 loading rate of 744 *μ*g/mg (RCGD423:MPDA, w/w) and RCGD423 at a feeding concentration of 0.4 mg/ml were used in subsequent experiments to measure the overall efficacy of MPDA as a drug carrier. In this regard, we measured UV/visible light absorption to determine the successful loading of RCGD423 into MPDA. Our results in this regard showed adsorption at 200–800 nm, with a significant RCGD423 peak at 200–240 nm in the carrier background [Fig. 3(b)]. Within 28 days, 83.9% of the loaded RCGD423 was released from the RCGD423@MPDA microspheres [Fig. 3(c)], and within 1 month from the initial incubation time, almost all RCGD423 had been released from the MPDA nanoparticles. The pattern of drug release over time was also found to be consistent.

We then evaluated whether the manufactured RCGD423@MPDA microspheres affected articular chondrocyte viability. The cell counting kit-8 (CCK-8) assay revealed that at concentrations of 1, 2, or 5 *μ*g/ml, MPDA nanoparticles did not significantly affect cell viability; however, when the concentration was increased to 10 and 20 *μ*g/ml, cell viability significantly decreased. RCGD423@MPDA, at 1, 2, and 5 *μ*g/ml improved cell viability relative to the control and MPDA-only treatments at the same concentrations [Figs. 3(d) and 3(e)]. In addition, EdU staining revealed that at 5 *μ*g/ml, RCGD423@MPDA increased the ratio of proliferating chondrocytes, whereas the MPDA-only treatment did not exert any significant effect on chondrocyte proliferation [Fig. 3(f)]. Therefore, in follow-up experiments, MPDA at concentrations of 1, 2, and 5 *μ*g/ml, with or without RCGD423, was employed.

### Prevention of IL-6-induced cartilage inflammation with RCGD423@MPDA

**FIG. 4.**
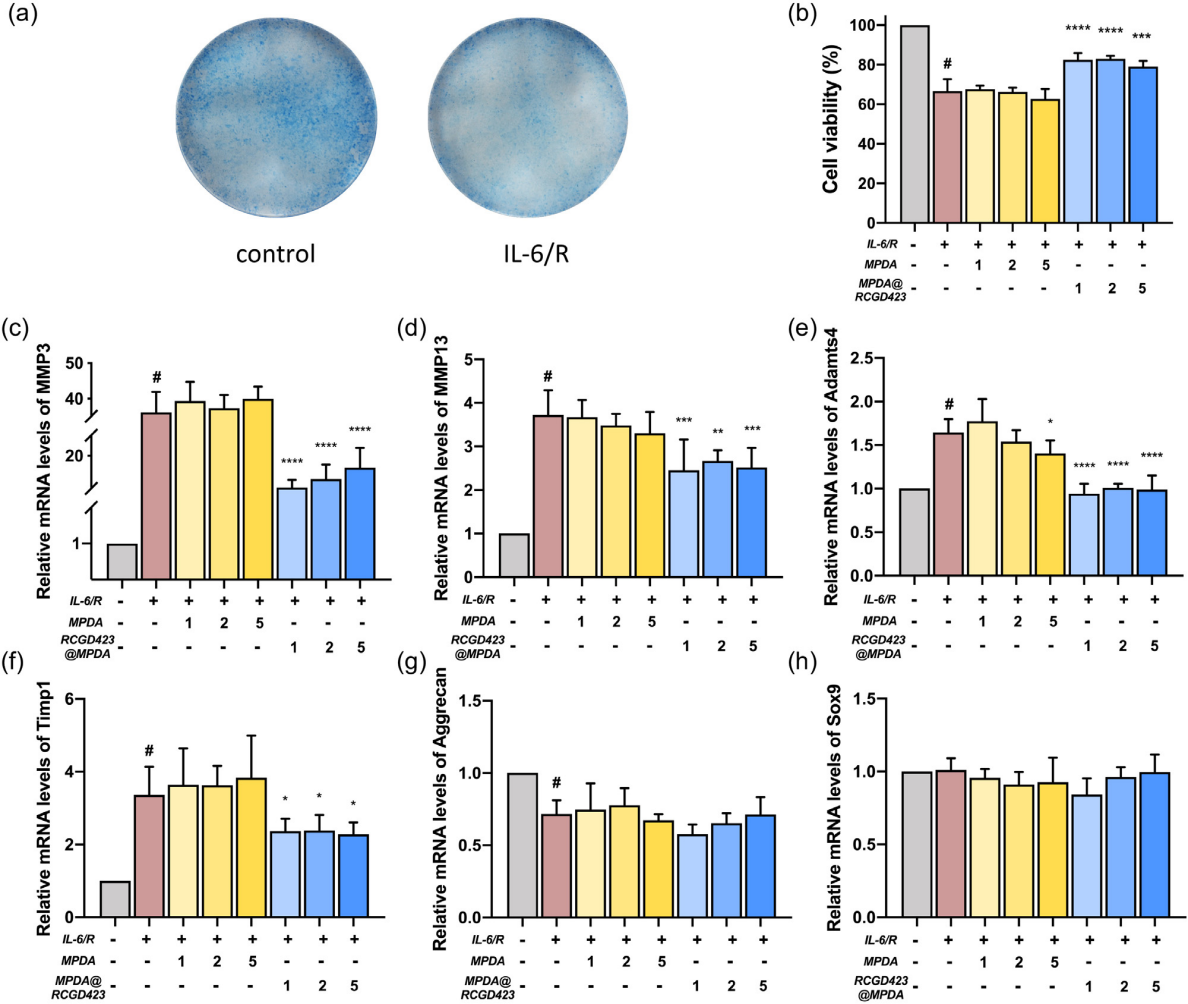
*In vitro* chondroprotective effect of RCGD423@MPDA nanoparticles. (a) Representative Alcian blue staining images corresponding to mouse chondrocytes treated (or not) with recombinant IL-6/IL-6R alpha complex (50 ng/ml) for 6 days. (b) Chondrocytes treated with a combination of IL-6/IL-6R and RCGD423@MPDA nanoparticles, IL-6/IL-6R (50 ng/ml) only, a combination of IL-6/IL-6R and MPDA nanoparticles, or not treated (control), for 48 h. Cell viability was assessed using the CCK-8 assay. (c)–(f) Chondrocytes co-treated with IL-6/IL-6R and MPDA nanoparticles for 48 h. The mRNA expression levels of *MMP3*, *MMP13*, *Adamts4*, and *Timp1* were determined using qPCR. (g) and (h) Chondrocytes co-treated with IL-6/IL-6R and MPDA nanoparticles for 48 h. The mRNA expression levels of *Acan* and *Sox9* were determined using qPCR. ^#^P < 0.05 vs control group; *P < 0.05, **P < 0.01, ***P < 0.001, and ****P < 0.0001, vs IL-6/IL-6R group.

Alcian blue staining showed that the glycosaminoglycan content in the extracellular matrix layer of the chondrocytes decreased after 6 days of IL-6 (50 ng/ml) stimulation [Fig. 4(a)], and based on the CCK-8 assay results, IL-6 treatment reduced chondrocyte viability, whereas the subsequent RCGD423@MPDA administration reversed this damage. This observation indicated that RCGD423@MPDA partially prevented IL-6-induced chondrocyte damage [Fig. 4(b)].

We investigated the effects of IL-6 on the expression levels of a disintegrin and metalloproteinase with thrombospondin-like motifs (ADAMTS), matrix metalloproteinases (MMP) and significant anabolic and phenotypic genes in murine chondrocytes. The incubation of the cells with IL-6 for 48 h resulted in an increase in the expression of Mmp3, Mmp13, Adamts4, and Timp1 mRNA [Figs. 4(c)–4(f)]. However, the addition of RCGD423@MPDA downregulated the expression of these genes. Unexpectedly, IL-6-induced Adamts4 upregulation was reversed by the 5 *μ*g/ml MPDA-only treatment. However, it did not significantly alter the IL-6-induced upregulation of the mRNA levels of other genes. Our results also indicated that IL-6 did not alter the mRNA expression levels of chondrogenic differentiation-related gene, *Sox9* [Figs. 4(g) and 4(h)], but upregulated that of the anabolic gene, *Acan*, which encodes Aggrecan. Furthermore, MPDA and RCGD423@MPDA did not significantly alter IL-6-induced *Acan* or *Sox9* expression.

### Cartilage apoptosis inhibitory effect of RCGD423@MPDA

**FIG. 5.**
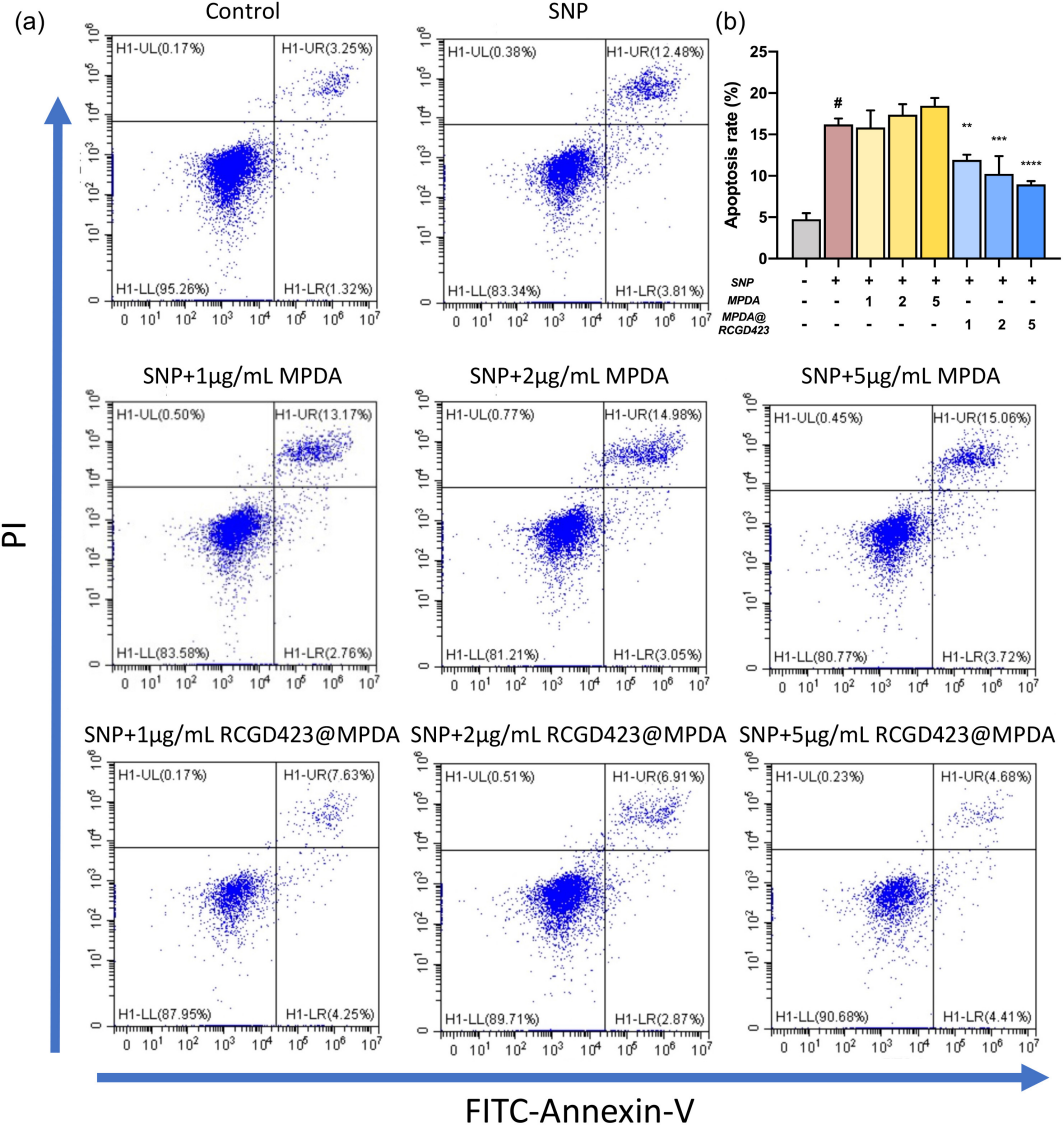
*In vitro* anti-apoptotic effect of RCGD423@MPDA nanoparticles. (a) Chondrocytes co-treated with 0.5 mM sodium nitroprusside (SNP) and MPDA nanoparticles, with or without RCGD423, for 24 h. Chondrocyte apoptosis was evaluated using flow cytometry. (b) Chondrocyte apoptosis rate. ^#^P < 0.05 vs control group; *P < 0.05, **P < 0.01, *** P < 0.001, and ****P < 0.0001, vs 0.5 mM SNP-treated group.

Flow cytometry revealed the occurrence of sodium nitroprusside (SNP) treatment-induced chondrocyte apoptosis [Figs. 5(a) and 5(b)], and even though both early apoptosis and late apoptosis were promoted, the latter was promoted to a greater extent. Specifically, when RCGD423@MPDA was combined with SNP, RCGD423@MPDA (at 5 *μ*g/ml) significantly reduced apoptosis, primarily late apoptosis. However, in the presence of SNP, the MPDA-only treatment did not alter apoptosis.

### *In vivo* chondroprotective effect of RCGD423@MPDA microspheres on drug-induced osteoarthritis in the rat model

**FIG. 6.**
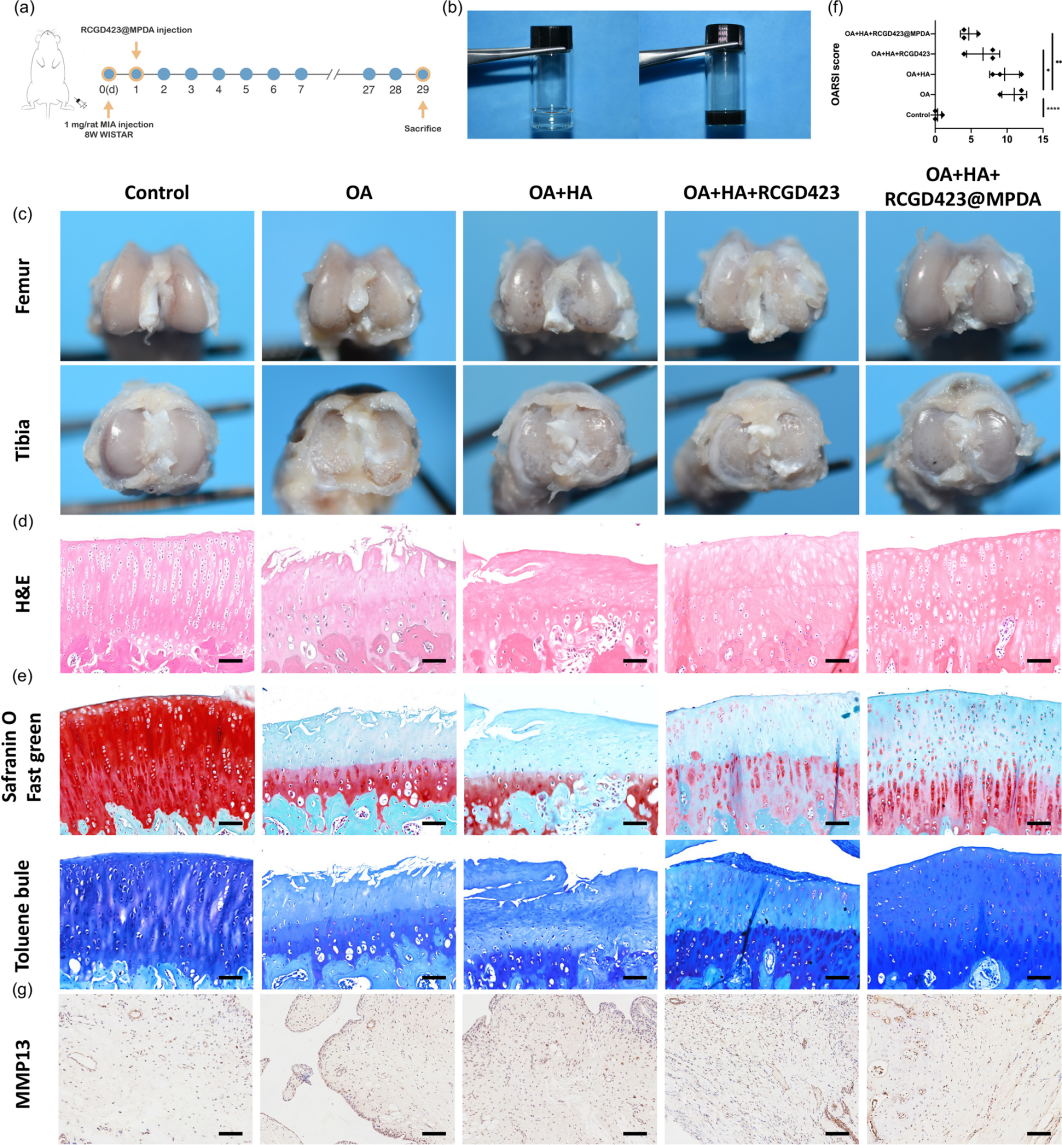
*In vivo* therapeutic effect of RCGD423@MPDA. (a) Schematic of the establishment of the rat osteoarthritis model and timing of intra-articular RCGD423@MPDA administration and animal sacrifice. (b) Hyaluronic acid and hyaluronic acid mixed with RCGD423@MPDA. (c) Macroscopic photographs of the knee joints; Scale bar = 100 *μ*m. (d) Hematoxylin and eosin staining images; Scale bar = 100 *μ*m. (e) Toluidine blue and Safranin O Fast Green staining images; Scale bar = 100 *μ*m. (f) OARSI scores calculated for three individuals in each group and presented as mean ± SD. (g) Matrix metalloproteinase 13 (MMP-13) immunohistochemical staining of synovial membrane cells; Scale bar = 100 *μ*m.

To further explore the potential effect of controlled RCGD423 release in osteoarthritis, we established a rat model of osteoarthritis by injecting MIA into the joint cavity. At 4 weeks after injection, knee-joint tissue was extracted, and histopathology and immunohistochemistry staining were performed [Figs. 6(a) and 6(b)]. The articular surface showed cartilage damage and surface irregularities owing to arthritis [Fig. 6(c)]. However, injecting RCGD423 + sodium hyaluronate (HA) and RCGD423@MPDA + HA into the joint cavity reduced cartilage deterioration to varying degrees. Specifically, in the osteoarthritis group, H&E staining showed a discontinuous joint cartilage surface, with eroded cartilage and an incomplete surface area loss [Fig. 6(d)]. Based on toluidine blue and safranin O staining, the osteoarthritis group showed a decrease in the glucosamine polysaccharide content compared with the control group [Fig. 6(e)], and the OARSI score also increased significantly [Fig. 6(f)]. Although a single HA treatment has a certain tendency to improve osteoarthritis, the overall damage of osteoarthritis does not improve significantly. This was ascertained by the lack of significant difference between the OA group and OA + HA group in terms of the OARSI score. Dispersing RCGD423 and RCGD423@MPDA in HA for intra-articular injection therapy can relatively delay their retention time in the joint cavity. Furthermore, the intra-articular injection of RCGD423@MPDA + HA prevented inflammation-induced joint erosion compared with that in the untreated osteoarthritis group, and the OARSI score corresponding to the treatment group was significantly lower than that in the untreated osteoarthritis group. In addition, RCGD423@MPDA + HA treatment resulted in better outcomes than the RCGD423 + HA-only treatment. These findings highlighted the beneficial effects of MPDA nanoparticles. We also investigated the biochemical changes in knee-joint composition via matrix metalloproteinase 13 (MMP-13) staining [Fig. 6(g)]. We observed that the MMP-13 content of knee-joint synovial membrane was significantly higher in the osteoarthritis group than in the control group. Our results also indicated a lower MMP-13 content in the hyaluronic acid, RCGD423 + HA and RCGD423@MPDA + HA treatment groups than in the untreated group.

## DISCUSSION

Developing a long-term treatment for osteoarthritis and delaying its progression remain challenging. Here, we introduced a new drug-clearance retardation system, derived from tumor-treating MPDA particles and involving the loading of small molecules into this biocompatible nanoscale MPDA. This system maintained a steady and controlled drug release for over 28 days and ensured inflammatory regulation. Thus, it achieved a longer-lasting effect than the non-vectored drug treatment.

Notably, MPDA is based on mussel-inspired polydopamine, which exhibits adhesive properties and improves adsorption.^19,20^ It has also been proved that MPDA provides considerably better drug loading than non-porous PDA nanomaterials or mesoporous silicon.^21,22^ In this study, the results indicated that the MPDA nanoparticles were characterized by irregular pores (diameter <5 nm) that enabled the formation of porous structures. Moreover, another peak of pore size observed at 23 nm (range 15–35 nm), indicated the presence of hollow cavities in the MPDA structure. Moreover, high-performance liquid chromatography (HPLC) results revealed that the drug was successfully loaded onto MPDA nanoparticles via adsorption. Specifically, the optimal RCGD423-loading capacity of MPDA was 744 *μ*g/mg, implying 93% encapsulation. MPDA drug loading typically reaches equilibrium after 48 h.^15^ However, differences in the distribution of mesoporous structures result in porous and non-porous polydopamine showing different drug-loading capacities. The patterns of PDA accumulation and interconnection generated slit-like mesopores in the MPDA structure. In addition, the aromatic ring structure of MPDA possibly led to π–π stacking as well as hydrophobic interactions with RCGD423 and subsequent multilayer adsorption, which potentially enhanced adsorption.^18^

Most of the loaded RCGD423 was released evenly over the 28-day observation period. Given that the drug-release rate of mesoporous materials increases with pore size,^23^ this moderate pace of sustained drug release was possibly related to the porous structure and pore size of MPDA. Moreover, the controlled release of RCGD423 may avoid the potential toxicity that can occur when high doses are administered locally. MPDA shows a high affinity for and retention of small molecules, help prevent their premature release. However, the stability of the loaded nanoparticles in warm solution is unknown. Therefore, it is possible that a portion of the loaded RCGD423 degraded before its concentration was measured. Furthermore, the actual amount of RCGD423 released may be greater than the observed amount.

To minimize the potential cytotoxicity of the microsphere system, we used the smallest tested dose. The relatively narrow range of safe concentrations of MPDA obtained in our experiments may be due to the fact that the murine primary chondrocytes are more sensitive to environmental changes than cell lines, and the chondrocytes in our study were also incubated with MPDA for longer periods. Thus, we observed that low-dose RCGD423@MPDA provided significant cartilage protection, and based on the CCK-8 assay, the cartilage cell proliferation and viability corresponding to MPDA doses of 1, 2, or 5 *μ*g/ml did not differ significantly from those of the control. This is consistent with previous findings and indicates that MPDA is not inherently toxic.^24,25^ Conversely, the same concentrations of RCGD423@MPDA exhibited a greater cartilage cell vitality-improvement effect. Therefore, MPDA-loaded drugs can exert their protective roles in promoting cell vitality, without interference from MPDA.

Inflammation and pro-inflammatory mediators play critical roles in osteoarthritis pathogenesis.^26,27^ Specifically, the roles of IL-6 family members in cartilage biology and pathogenesis have been investigated.^11,28,29^ IL-6, Oncostatin-M (OSM), leukemia inhibitory factor (LIF), and other IL-6 family members promote osteoarthritis, either as inflammatory factors or by directly regulating matrix destruction. When bound to its specific receptor, IL-6R, IL-6 activates a homodimer of the signal-transducing β-receptor, gp130, which is shared by all IL-6 family members.^30^ Thus, we loaded the recently systematically validated small molecule, RCGD423, an anti-inflammatory drug, in our system.^9^ As a new small-molecule gp130 modulator, RCGD423 substantially attenuated cartilage damage in our rat model. Specifically, it promoted signaling by promoting the formation of active homologous dimers, primarily via interactions with pSTAT3/MYC. This actively counteracted IL-6 family cytokine-mediated isomerization, thereby inhibiting hypertrophy as well as decomposition mediated by ERK1/2 and NF-κB.^9^ Although MPDA had no effect on the inflammatory process in our *in vitro* experiments, it possibly played a role in reversing the previously reported cytokine-induced inflammation that caused cartilage degradation and apoptosis.^31^ Indeed, this microsphere-carrying system reduced osteoarthritis damage by reducing the production of inflammation-related apoptosis and degradation-related factors, without significantly affecting cartilage synthesis. In addition, *in vivo*, the RCGD423@MPDA system showed better efficacy than the other treatments (control, hyaluronic acid only, and hyaluronic acid combined with RCGD423 without MPDA). Collectively, these findings confirmed that the long-term treatment of osteoarthritis using MPDA can delay the progression of the disease. Thus, we provide evidence of the effectiveness of an MPDA-based controlled-release drug-loading system. These results could serve as a reference for designing and preparing a stable microsphere-loading system.

## CONCLUSIONS

The results of this study indicated that this microsphere drug-clearance retardation system, with MPDA as the carrier, has a considerable drug-loading capacity; this system offers the possibility to effectively improve degenerative changes within arthritic cartilage. Furthermore, as a vector, MPDA shows a longer drug-retention time than previously used poly(lactic-co-glycolic acid) carriers and also provides more stable and long-term drug release. Therefore, by prolonging drug-release cycles, the required frequency of intra-joint drug injection can be substantially reduced. This study also provides insights into the selection of drug-clearance retarding vectors and highlights the applicability of MPDA in this regard.

## METHODS

### Synthesis of MPDA nanoparticles

MPDA nanoparticles were prepared as previously described.^32^ Briefly, dopamine hydrochloride (0.30 g) and F127 (0.2 g) were dissolved in a mixed solution containing de-ionized water (10 ml) and ethanol (10 ml) while stirring. After 30 min, trimethyl benzene (320 *μ*l) was added, and the mixture was sonicated for 10 min in a water bath. Subsequently, 750 *μ*l of ammonia solution was added dropwise while stirring. Next, the reaction mixture was stirred at room temperature for 2 h, and finally, MPDA nanoparticles were obtained by centrifugation at 13 000 × *g* for 15 min, washed several times with water and ethanol, and suspended in water for further use. The morphological characteristics of the MPDA nanoparticles were determined using scanning electron microscopy (SEM, ZEISS GeminiSEM 300, Germany) and transmission electron microscopy (TEM, FEI Talos S-FEG, USA), and their surface parameters were measured from N_2_ adsorption/desorption isotherms obtained using an ASAP 2460 Surface Area and Porosity Analyzer (Micromeritics Instrument Corp., Norcross, GA, USA).

### Synthesis of the RCGD423@MPDA system

MPDA (5 mg) was added to 10 ml of phosphate-buffered saline (PBS; pH = 7.4; Gibco, Grant Island, NY, USA) containing 0.4 mg/ml RCGD423 (MedChemExpress, Monmouth Junction, NJ, USA). Thereafter, the mixture was stirred on ice for 48 h. This was followed by centrifugation to collect the precipitated nanoparticles. RCGD423 was quantified using high-performance liquid chromatography (HPLC), and by subtracting the quantity of RCGD423 in the supernatant from its total quality in the initial solution, the loading capacity of MPDA was determined. Furthermore, UV-visible spectrometry was used to determine that RCGD423 was already loaded into the MPDA.

### Drug-release experiments

First, 0.5 mg of RCGD423-loaded nanoparticles was dispersed in PBS (1 ml; pH = 7.4) on a low-speed shaker at approximately 37 °C. Thereafter, at predetermined intervals (1, 4, 7, 10, 14, 21, and 28 days), 1 ml of aliquots was withdrawn from the Eppendorf tubes and the quantity of the released RCGD423 was determined using HPLC (an RCGD423 standard curve was generated, and the figure obtained was analyzed with reference to the standard curve). To maintain a constant volume, 1 ml of fresh medium was added after each sampling. The concentration and content of the specimen were calculated according to the HPLC results, and the cumulative release of RCGD423 over a certain period was then determined. Finally, the quantities of RCGD423 released were averaged for triplicate measurements.

### Cell culture

Murine chondrocytes were isolated as previously described.^33^ Thereafter, they were cultured in Dulbecco's modified Eagle media-F12 (Gibco) supplemented with 10% fetal bovine serum (Gibco), 100 U/ml penicillin, and 100 U/ml streptomycin. The chondrocytes were cultured at 37 °C in a humidified incubator containing 5% CO_2_. When needed, chondrocytes were stimulated with 50 ng/ml Mouse IL-6/IL-6R Alpha Complex (R&D Systems, Minneapolis, MN, USA).

### Cytotoxicity assay

Cell counting kit-8 (CCK-8) assay was performed to evaluate the cytotoxicity and viability of murine articular chondrocytes. Briefly, chondrocytes were seeded in 96-well plates at a cell density of 5 × 10^3^ cells per well and incubated at 37 °C for 24 h. Next, the culture medium was replaced with fresh medium only or the fresh medium containing various concentrations of MPDA, MPDA@RCGD423. After incubation for 48 h, the medium was removed, and 10 *μ*l of CCK-8 (Dojindo, Kumamoto, Japan) and 100 *μ*l of fresh medium were added, followed by further incubation for 3 h. Finally, absorbance of the samples was measured at 450 nm using a microplate reader (SpectraMax i3x; Molecular Devices, Sunnyvale, CA, USA).

### 5-Ethynyl-2′-deoxyuridine (EdU) staining

EdU staining was performed to evaluate chondrocyte proliferation. Briefly, 5 × 10^3^ chondrocytes were inoculated in 96-well plates. After 24 h, the drug-loaded nanoparticles together with the cartilage cells were added and the mixture was incubated at 37 °C for another 24 h, followed by the addition of EdU dye. After 2 h, the medium was discarded, and the cells were fixed with 4% polyformaldehyde solution. In the next step, Apollo reaction solution and Hoechst 33342 (Ribobio, Guangzhou, China) were added to the plates in the dark. After 30 min, the cells were washed with PBS and images were captured using an inverted fluorescence microscope (ZEISS, Germany).

### Quantitative real-time polymerase chain reaction (qPCR)

After co-culturing with RCGD423 and IL-6 (50 ng/ml) for 48 h, total cell mRNA was extracted using the RNAiso Plus Kit (Takara, Shiga, Japan). Thereafter, cDNA was reverse-transcribed using Reverse Transcription Master Mix (EZBioscience, Roseville, MN, USA). All PCRs were then performed with reaction mixtures of volume 20 *μ*l using an asymmetrical cyanine dye used as a nucleic acid stain in molecular biology SYBR Green qPCR Master Mix (ROX2 plus) (EZBioscience). Glyceraldehyde-3-phosphate dehydrogenase (GAPDH) was used as an internal control for PCR amplification, and the expression levels of inflammatory markers, ADAMTS-4, MMP-3, MMP-13, and Timp1, as well as those of synthetic-related markers, including Acan and Sox9, were determined.

### Flow cytometry

Chondrocytes (1 × 10^5^ cells/ml) were plated in a six-well plate. When the cells reached 75%–85% confluence, 5% serum culture containing 0.5 mmol/l sodium nitroprusside (SNP), with or without nanoparticles, was added to the cells. The SNP was added to induce apoptosis and clarify the effect of RCGD423@MPDA on murine chondrocytes.^34^ After 24 h, the upper liquid and cells were collected and centrifuged at 400 × *g* for 5 min. The supernatant was removed, and PBS (2 ml) was added to each centrifuge tube. After cleaning, the samples were again centrifuged at 400* × g* for 5 min. Thereafter, PBS was removed, and the cells were resuspended in 100 *μ*l of 1× binding buffer and transferred into flow cytometry tubes. Next, 5 *μ*l of annexin V-FITC dye (BD Biosciences, Franklin Lakes, NJ, USA) and 5 *μ*l of propidium iodide dye were added to the tubes; then, the mixtures were gently mixed for 15 min at room temperature (approximately 25 °C). Finally, 400 *μ*l of 1× binding buffer was added, and the proportion of apoptotic cells was determined for each group of murine cartilage cells using flow cytometry.

### Animal experiments

Male Wistar rats (age, 8 weeks; weight, 300–320 g), purchased from Vital River Laboratory Animal Technology Co., Ltd. (Beijing, China), were placed in a room with normal lighting (12-h/12-h light/dark cycle) at 25 °C before they were sacrificed. The rats were randomly allocated to five groups: control (n = 6), osteoarthritis (n = 6), osteoarthritis + sodium hyaluronate (HA) (n = 6), osteoarthritis + HA + RCGD423 (n = 6), and osteoarthritis + HA + RCGD423@MPDA (n = 6). The nanoparticles were dissolved in 0.5% HA. Furthermore, to establish the osteoarthritis model, a single intra-articular injection of 1 mg monosodium iodoacetate (MIA, Sigma, St. Louis, MO, USA) was administered to the rats in the treatment groups, whereas the control rats were injected an equal volume of saline. In all treatments, RCGD423 was administered at a dose of 20 *μ*g/rat, 1 day after MIA injection.

### Histopathological staining

Three rats in each group were used for gross morphology observations, and the other three rats were used for histological analysis. The animals were euthanized via cervical dislocation at 4 weeks post-treatment, and their entire knee joints were removed, fixed in 4% paraformaldehyde at room temperature for 24 h, and thereafter, decalcified using 10% ethylenediaminetetraacetic acid solution at 37 °C for 5 weeks. After the knee joints were dehydrated and embedded in paraffin, serial 5-*μ*m-thick sections were cut sagittally from the knee joints and stained with hematoxylin and eosin (H&E), toluidine blue, and Safranin O Fast Green. Osteoarthritis severity was then assessed using the Osteoarthritis Research Society International (OARSI) scoring system.^35^

### Immunohistochemical staining

After dewaxing and rehydrating knee-joint slices, they were incubated in a water bath at 98 °C for 30 min and fixed in sodium citrate solution. When the slices reached room temperature, 3% hydrogen peroxide was added to eliminate endogenous peroxidase. This was followed by incubation in PBS containing 10% goat serum for 30 min to block nonspecific antigens. Next, MMP-13 antibody (8165-1-AP, 1:100, Proteintech, Rosemont, IL, USA) was added, and the sections were placed in a wet box overnight at 4 °C. Thereafter, secondary antibody was added followed by incubation for 30 min at room temperature. Finally, the slices were visualized using a diaminobenzidine kit, and photographs were taken using an optical microscope.

### Statistical analysis

Data were analyzed using the one-way analysis of variance followed by Dunnett's test. All statistical analyses were performed using GraphPad Prism software v8.4.0 (GraphPad, Inc., San Diego, CA, USA), and statistical significance was set at P < 0.05. The results are presented as mean ± SD.

## SUPPLEMENTARY MATERIAL

See the supplementary material for XRD pattern of MPDA and immunofluorescence staining of chondrocytes. The table includes primers of targeted genes.

## Data Availability

The data that support the findings of this study are available within the article and its supplementary material.
